# Supplementary material for: Canadian Expert Consensus Recommendations for the Diagnosis and Management of Glioblastoma: Results of a Delphi Study
Source: Curr Oncol. 2025 Apr 1;32(4):207. doi: 10.3390/curroncol32040207 (PMC12026134; doi:10.3390/curroncol32040207)
Supplement: Supplementary file 1 [file curroncol-32-00207-s001.zip › curroncol-3524934-supplementary.pdf]

Supplementary Materials

Table S1. Members of the consensus-building initiative.

Expert Name	Province	Discipline/Specialties
Warren P. Mason	Ontario	Neuro-Oncologist
Rebecca A. Harrison	British Columbia	Neuro-Oncologist
Sarah Lapointe	Quebec	Neuro-Oncologist
Mary Jane Lim-Fat	Ontario	Neuro-Oncologist
Mary V. MacNeil	Nova Scotia	Medical Oncologist
David Mathieu	Quebec	Neurosurgeon
James R. Perry	Ontario	Neuro-Oncologist
Marshall W. Pitz	Manitoba	Medical Oncologist
David Roberge	Quebec	Radiation Oncologist
Derek S. Tsang	Ontario	Radiation Oncologist
Christina Tsien	Quebec	Radiation Oncologist
Frank K. H. van Landeghem	Alberta	Neuropathologist
Gelareh Zadeh	Ontario	Neurosurgeon
Jacob Easaw	Alberta	Medical Oncologist

Table S2. List of research articles and guidelines reviewed before drafting the statements submitted to the Delphi process.

No.	Reference
1	Hambardzumyan D, Bergers G. Glioblastoma: defining tumor niches. *Trends Cancer*. Dec 2015;1(4):252-265. doi:10.1016/j.trecan.2015.10.009
2	Hanif F, Muzaffar K, Perveen K, Malhi SM, Simjee Sh U. Glioblastoma multiforme: a review of its epidemiology and pathogenesis through clinical presentation and treatment. *Asian Pac J Cancer Prev*. Jan 1 2017;18(1):3-9. doi:10.22034/APJCP.2017.18.1.3
3	Walker E, Zakaria D, Yuan Y, Yasmin F, Shaw A, Davis F. Brain Tumour Registry of Canada (BTRC): Incidence (2013-2017) and Mortality (2014-2018) Report. Brain Tumour Registry of Canada (BTRC) A Surveillance Research Collaborative. 2021. https://braintumourregistry.ca/incidence-report
4	Easaw JC, Mason WP, Perry J, et al. Canadian recommendations for the treatment of recurrent or progressive glioblastoma multiforme. *Curr Oncol*. Jun 2011;18(3):e126-36. doi:10.3747/co.v18i3.755
5	Mason WP, Maestro RD, Eisenstat D, et al. Canadian recommendations for the treatment of glioblastoma multiforme. *Curr Oncol*. Jun 2007;14(3):110-7. doi:10.3747/co.2007.119
6	Berger TR, Wen PY, Lang-Orsini M, Chukwueke UN. World Health Organization 2021 classification of central nervous system tumors and implications for therapy for adult-type gliomas: a review. *JAMA Oncol*. Oct 1 2022;8(10):1493-1501. doi:10.1001/jamaoncol.2022.2844
7	Walker E, Liu J, Davis F, Climans S, Yuan Y. *Brain Tumour Registry of Canada*
8	(BTRC): Survival and Prevalence Report 2010–2017. Brain Tumour Registry of Canada (BTRC) A Surveillance Research Collaborative. 2022. https://braintumourregistry.ca/2022-survival-and-prevalence-report
9	Komlodi-Pasztor E, Blakeley JO. Brain cancers in genetic syndromes. *Curr Neurol Neurosci Rep*. Nov 22 2021;21(11):64. doi:10.1007/s11910-021-01149-4
10	Johansson G, Andersson U, Melin B. Recent developments in brain tumor predisposing syndromes. *Acta Oncol*. 2016;55(4):401-11. doi:10.3109/0284186X.2015.1107190
11	Tamimi AF, Juweid M. Epidemiology and outcome of glioblastoma. In: De Vleeschouwer S, ed. *Glioblastoma*. 2017.
12	Braganza MZ, Kitahara CM, Berrington de Gonzalez A, Inskip PD, Johnson KJ, Rajaraman P. Ionizing radiation and the risk of brain and central nervous system tumors: a systematic review. *Neuro Oncol*. Nov 2012;14(11):1316-24. doi:10.1093/neuonc/nos208
13	Kiang KM, Sun S, Leung GK. ADD3 deletion in glioblastoma predicts disease status and survival. *Front Oncol*. 2021;11:717793. doi:10.3389/fonc.2021.717793
14	Taylor OG, Brzozowski JS, Skelding KA. Glioblastoma multiforme: an overview of emerging therapeutic targets. *Front Oncol*. 2019;9:963. doi:10.3389/fonc.2019.00963
15	Louis DN, Perry A, Wesseling P, et al. The 2021 WHO classification of tumors of the central nervous system: a summary. *Neuro Oncol*. Aug 2 2021;23(8):1231-1251. doi:10.1093/neuonc/noab106
16	Xia L, Wu B, Fu Z, et al. Prognostic role of IDH mutations in gliomas: a meta-analysis of 55 observational studies. *Oncotarget*. Jul 10 2015;6(19):17354-65. doi:10.18632/oncotarget.4008

17	Gibson D, Ravi A, Rodriguez E, et al. Quantitative analysis of MGMT promoter methylation in glioblastoma suggests nonlinear prognostic effect. *Neurooncol Adv*. Jan-Dec 2023;5(1):vdad115. doi:10.1093/noajnl/vdad115
18	Hegi ME, Liu L, Herman JG, et al. Correlation of O6-methylguanine methyltransferase (MGMT) promoter methylation with clinical outcomes in glioblastoma and clinical strategies to modulate MGMT activity. *J Clin Oncol*. Sep 1 2008;26(25):4189-99. doi:10.1200/JCO.2007.11.5964
19	Szylberg M, Sokal P, Sledzinska P, et al. MGMT promoter methylation as a prognostic factor in primary glioblastoma: a single-institution observational study. *Biomedicines*. Aug 20 2022;10(8)doi:10.3390/biomedicines10082030
20	Woo HY, Na K, Yoo J, et al. Glioblastomas harboring gene fusions detected by next-generation sequencing. *Brain Tumor Pathol*. Oct 2020;37(4):136-144. doi:10.1007/s10014-020-00377-9
21	Le Rhun E, Preusser M, Roth P, et al. Molecular targeted therapy of glioblastoma. *Cancer Treat Rev*. Nov 2019;80:101896. doi:10.1016/j.ctrv.2019.101896
22	McKinnon C, Nandhabalan M, Murray SA, Plaha P. Glioblastoma: clinical presentation, diagnosis, and management. *BMJ*. Jul 14 2021;374:n1560. doi:10.1136/bmj.n1560
23	Gilard V, Tebani A, Dabaj I, et al. Diagnosis and management of glioblastoma: a comprehensive perspective. *J Pers Med*. Apr 1 2021;11(4)doi:10.3390/jpm11040258
24	M IJ-K, Snijders TJ, de Graeff A, Teunissen S, de Vos FYF. Prevalence of symptoms in glioma patients throughout the disease trajectory: a systematic review. *J Neurooncol*. Dec 2018;140(3):485-496. doi:10.1007/s11060-018-03015-9
25	Ferreira LP, Pinheiro CF, Fernandes NA, Ferreira CN. When depression hides a brain tumor: a case of glioblastoma. *Prim Care Companion CNS Disord*. Nov 14 2019;21(6)doi:10.4088/PCC.19l02455
26	Munjal S, Pahlajani S, Baxi A, Ferrando S. Delayed diagnosis of glioblastoma multiforme presenting with atypical psychiatric symptoms. *Prim Care Companion CNS Disord*. Dec 29 2016;18(6)doi:10.4088/PCC.16l01972
27	Leo RJ, Frodey JN, Ruggieri ML. Subtle neuropsychiatric symptoms of glioblastoma multiforme misdiagnosed as depression. *BMJ Case Rep*. Mar 17 2020;13(3)doi:10.1136/bcr-2019-233208
28	Young JS, Al-Adli N, Sibih YE, et al. Recognizing the psychological impact of a glioma diagnosis on mental and behavioral health: a systematic review of what neurosurgeons need to know. *J Neurosurg*. Jul 1 2023;139(1):11-19. doi:10.3171/2022.9.JNS221139
29	Solanki C, Sadana D, Arimappamagan A, et al. Impairments in quality of life and cognitive functions in long-term survivors of glioblastoma. *J Neurosci Rural Pract*. Apr-Jun 2017;8(2):228-235. doi:10.4103/0976-3147.203829
30	Amidei C, Kushner DS. Clinical implications of motor deficits related to brain tumors(dagger). *Neurooncol Pract*. Dec 2015;2(4):179-184. doi:10.1093/nop/npv017
31	Au TH, Willis C, Reblin M, et al. Caregiver burden by treatment and clinical characteristics of patients with glioblastoma. *Support Care Cancer*. Feb 2022;30(2):1365-1375. doi:10.1007/s00520-021-06514-0
32	Shi C, Lamba N, Zheng LJ, et al. Depression and survival of glioma patients: a systematic review and meta-analysis. *Clin Neurol Neurosurg*. Sep 2018;172:8-19. doi:10.1016/j.clineuro.2018.06.016
33	Applebaum AJ, Baser RE, Roberts KE, et al. Meaning-centered psychotherapy for cancer caregivers: a pilot trial among caregivers of patients with glioblastoma multiforme. *Transl Behav Med*. Aug 17 2022;12(8):841-852. doi:10.1093/tbm/ibac043
34	Carrano A, Zarco N, Phillipps J, et al. Human cerebrospinal fluid modulates pathways promoting glioblastoma malignancy. *Front Oncol*. 2021;11:624145. doi:10.3389/fonc.2021.624145
35	Ripari LB, Norton ES, Bodoque-Villar R, et al. Glioblastoma proximity to the lateral ventricle alters neurogenic cell populations of the subventricular zone. *Front Oncol*. 2021;11:650316. doi:10.3389/fonc.2021.650316
36	Rajagopalan V, El Kamar FG, Thayaparan R, Grossbard ML. Bone marrow metastases from glioblastoma multiforme – a case report and review of the literature. *J Neurooncol*. Apr 2005;72(2):157-61. doi:10.1007/s11060-004-3346-y
37	Mourad PD, Farrell L, Stamps LD, Chicoine MR, Silbergeld DL. Why are systemic glioblastoma metastases rare? Systemic and cerebral growth of mouse glioblastoma. *Surg Neurol*. Jun 2005;63(6):511-9; discussion 519. doi:10.1016/j.surneu.2004.08.062
38	Konishi Y, Muragaki Y, Iseki H, Mitsuhashi N, Okada Y. Patterns of intracranial glioblastoma recurrence after aggressive surgical resection and adjuvant management: retrospective analysis of 43 cases. *Neurol Med Chir (Tokyo)*. 2012;52(8):577-86. doi:10.2176/nmc.52.577
39	Rapp M, Baernreuther J, Turowski B, Steiger HJ, Sabel M, Kamp MA. Recurrence pattern analysis of primary glioblastoma. *World Neurosurg*. Jul 2017;103:733-740. doi:10.1016/j.wneu.2017.04.053
40	Kim HR, Kim KH, Kong DS, et al. Outcome of salvage treatment for recurrent glioblastoma. *J Clin Neurosci*. Mar 2015;22(3):468-73. doi:10.1016/j.jocn.2014.09.018
41	Smith T, Yuan Y, Walker E, Davis F. Brain Tumour Registry of Canada (BTRC): Survival Report 2010-2015. Brain Tumour Registry of Canada (BTRC) A Surveillance Research Collaborative. 2019. https://braintumourregistry.ca/survival-report
42	Efremov L, Abera SF, Bedir A, Vordermark D, Medenwald D. Patterns of glioblastoma treatment and survival over a 16-years period: pooled data from the German Cancer Registries. *J Cancer Res Clin Oncol*. Nov 2021;147(11):3381-3390. doi:10.1007/s00432-021-03596-5

43	Zhang K, Wang XQ, Zhou B, Zhang L. The prognostic value of MGMT promoter methylation in glioblastoma multiforme: a meta-analysis. *Fam Cancer*. Sep 2013;12(3):449-58. doi:10.1007/s10689-013-9607-1
44	Dai Y, Ning X, Han G, Li W. Assessment of the association between isocitrate dehydrogenase 1 mutation and mortality risk of glioblastoma patients. *Mol Neurobiol*. Apr 2016;53(3):1501-1508. doi:10.1007/s12035-015-9104-7
45	Yang P, Zhang W, Wang Y, et al. IDH mutation and MGMT promoter methylation in glioblastoma: results of a prospective registry. *Oncotarget*. Dec 1 2015;6(38):40896-906. doi:10.18632/oncotarget.5683
46	Gao H, Jiang X. Progress on the diagnosis and evaluation of brain tumors. *Cancer Imaging*. Dec 11 2013;13(4):466-81. doi:10.1102/1470-7330.2013.0039
47	Lundy P, Domino J, Ryken T, et al. The role of imaging for the management of newly diagnosed glioblastoma in adults: a systematic review and evidence-based clinical practice guideline update. *J Neurooncol*. Nov 2020;150(2):95-120. doi:10.1007/s11060-020-03597-3
48	Padelli F, Mazzi F, Erbetta A, et al. In vivo brain MR spectroscopy in gliomas: clinical and pre-clinical chances. *Clinical and Translational imaging*. 2022;10(5):495-515.
49	Bagley SJ, Schwab RD, Nelson E, et al. Histopathologic quantification of viable tumor versus treatment effect in surgically resected recurrent glioblastoma. *J Neurooncol*. Jan 2019;141(2):421-429. doi:10.1007/s11060-018-03050-6
50	Pasquini L, Napolitano A, Tagliente E, et al. Deep learning can differentiate IDH-mutant from IDH-wild GBM. *J Pers Med*. Apr 9 2021;11(4)doi:10.3390/jpm11040290
51	Richardson TE, Yokoda RT, Rashidipour O, et al. Mismatch repair protein mutations in isocitrate dehydrogenase (IDH)-mutant astrocytoma and IDH-wild-type glioblastoma. *Neurooncol Adv*. 2023;5(1):vdad085. doi:10.1093/noajnl/vdad085
52	Diachun LL, Klages KB, Hansen KT, Blake K, Gordon J. The comprehensive geriatric assessment guide: an exploratory analysis of a medical trainee performance evaluation tool. *Acad Med*. Dec 2012;87(12):1679-84. doi:10.1097/ACM.0b013e318271cad8
53	Lutgendorf-Caucig C, Freyschlag C, Masel EK, Marosi C. Guiding Treatment Choices for Elderly Patients with Glioblastoma by a Comprehensive Geriatric Assessment. *Curr Oncol Rep*. Jul 10 2020;22(9):93. doi:10.1007/s11912-020-00951-6
54	Chahal M, Thiessen B, Mariano C. Treatment of older adult patients with glioblastoma: moving towards the inclusion of a comprehensive geriatric assessment for guiding management. *Curr Oncol*. Jan 14 2022;29(1):360-376. doi:10.3390/curroncol29010032
55	Giosa JL, Stolee P, Holyoke P. Development and testing of the Geriatric Care Assessment Practices (G-CAP) survey. *BMC Geriatr*. Apr 1 2021;21(1):220. doi:10.1186/s12877-021-02073-5
56	Janjua TI, Rewatkar P, Ahmed-Cox A, et al. Frontiers in the treatment of glioblastoma: past, present and emerging. *Adv Drug Deliv Rev*. Apr 2021;171:108-138. doi:10.1016/j.addr.2021.01.012
57	Brown TJ, Brennan MC, Li M, et al. Association of the extent of resection with survival in glioblastoma: a systematic review and meta-analysis. *JAMA Oncol*. Nov 1 2016;2(11):1460-1469. doi:10.1001/jamaoncol.2016.1373
58	Revilla-Pacheco F, Rodriguez-Salgado P, Barrera-Ramirez M, et al. Extent of resection and survival in patients with glioblastoma multiforme: systematic review and meta-analysis. *Medicine (Baltimore)*. Jun 25 2021;100(25):e26432. doi:10.1097/MD.0000000000026432
59	Polonara G, Aiudi D, Iacoangeli A, et al. Glioblastoma: a retrospective analysis of the role of the maximal surgical resection on overall survival and progression-free survival. *Biomedicines*. 2023;11(3)doi:10.3390/biomedicines11030739
60	Fernandes C, Costa A, Osorio L, et al. Current standards of care in glioblastoma therapy. In: De Vleeschouwer S, ed. *Glioblastoma*. 2017.
61	Zhang M, Xu F, Ni W, et al. Survival impact of delaying postoperative chemoradiotherapy in newly diagnosed glioblastoma patients. *Transl Cancer Res*. Sep 2020;9(9):5450-5458. doi:10.21037/tcr-20-1718
62	Stupp R, Hegi ME, Mason WP, et al. Effects of radiotherapy with concomitant and adjuvant temozolomide versus radiotherapy alone on survival in glioblastoma in a randomised phase III study: 5-year analysis of the EORTC-NCIC trial. *Lancet Oncol*. May 2009;10(5):459-66. doi:10.1016/S1470-2045(09)70025-7
63	Stupp R, Mason WP, van den Bent MJ, et al. Radiotherapy plus concomitant and adjuvant temozolomide for glioblastoma. *N Engl J Med*. 2005;352(10):987-96. doi:10.1056/NEJMoa043330
64	Hegi ME, Diserens AC, Gorlia T, et al. MGMT gene silencing and benefit from temozolomide in glioblastoma. *N Engl J Med*. Mar 10 2005;352(10):997-1003. doi:10.1056/NEJMoa043331
65	Wang Y, Feng Y. The efficacy and safety of radiotherapy with adjuvant temozolomide for glioblastoma: a meta-analysis of randomized controlled studies. *Clin Neurol Neurosurg*. Sep 2020;196:105890. doi:10.1016/j.clineuro.2020.105890
66	Gupta T, Selvarajan JMP, Kannan S, Menon N, Dasgupta A, Chatterjee A. Updated systematic review and meta-analysis of extended adjuvant temozolomide in patients with newly diagnosed glioblastoma. *Neurooncol Adv*. Jan-Dec 2023;5(1):vdad086. doi:10.1093/noajnl/vdad086
67	Attarian F, Taghizadeh-Hesary F, Fanipakdel A, et al. A systematic review and meta-analysis on the number of adjuvant temozolomide cycles in newly diagnosed glioblastoma. *Front Oncol*. 2021;11:779491. doi:10.3389/fonc.2021.779491

68	Herrlinger U, Tzaridis T, Mack F, et al. Lomustine-temozolomide combination therapy versus standard temozolomide therapy in patients with newly diagnosed glioblastoma with methylated MGMT promoter (CeTeG/NOA-09): a randomised, open-label, phase 3 trial. *Lancet*. 2019;393(10172):678-688. doi:10.1016/S0140-6736(18)31791-4
69	Weller J, Zeyen T, Schlegel U, et al. Lomustine/temozolomide chemotherapy for newly diagnosed MGMT-methylated IDHwt glioblastoma according to CeTeG/NOA–09: real-world experience in a multicenter cohort. *Neuro-Oncology*. 2022;24(Supplement_7):vii71-vii71. doi:10.1093/neuonc/noac209.274
70	Ram Z, Kim CY, Hottinger AF, Idbaih A, Nicholas G, Zhu JJ. Efficacy and safety of tumor treating fields (TTFields) in elderly patients with newly diagnosed glioblastoma: subgroup analysis of the phase 3 EF-14 clinical trial. *Front Oncol*. 2021;11:671972. doi:10.3389/fonc.2021.671972
71	Stupp R, Taillibert S, Kanner A, et al. Effect of Tumor-Treating Fields Plus Maintenance Temozolomide vs Maintenance Temozolomide Alone on Survival in Patients With Glioblastoma: A Randomized Clinical Trial. *JAMA*. Dec 19 2017;318(23):2306-2316. doi:10.1001/jama.2017.18718
72	Ballo MT, Conlon P, Lavy-Shahaf G, Kinzel A, Vymazal J, Rulseh AM. Association of tumor treating fields (TTFields) therapy with survival in newly diagnosed glioblastoma: a systematic review and meta-analysis. *J Neurooncol*. Aug 2023;164(1):1-9. doi:10.1007/s11060-023-04348-w
73	Oster C, Schmidt T, Agkatsev S, et al. Are we providing best-available care to newly diagnosed glioblastoma patients? Systematic review of phase III trials in newly diagnosed glioblastoma 2005-2022. *Neurooncol Adv*. Jan-Dec 2023;5(1):vdad105. doi:10.1093/noajnl/vdad105
74	Nishikawa R, Yamasaki F, Arakawa Y, et al. Safety and efficacy of tumour-treating fields (TTFields) therapy for newly diagnosed glioblastoma in Japanese patients using the Novo-TTF System: a prospective post-approval study. *Jpn J Clin Oncol*. Apr 29 2023;53(5):371-377. doi:10.1093/jjco/hyad001
75	Canada's Drug and Health Technology Agency (CADHT). Alternating electric fields ("tumour-treating fields") for the treatment of glioblastoma. 2018. Accessed February 9, 2024. https://www.cadth.ca/sites/default/files/pdf/EH0060_alternating_electric_fields_for_the_treatment_of_glioblastoma.pdf
76	Connock M, Auguste P, Dussart C, Guyotat J, Armoiry X. Cost-effectiveness of tumor-treating fields added to maintenance temozolomide in patients with glioblastoma: an updated evaluation using a partitioned survival model. *J Neurooncol*. Jul 2019;143(3):605-611. doi:10.1007/s11060-019-03197-w
77	NCCN Clinical Practice Guidelines in Oncology. Central nervous system cancers. Version 1.2023. 2023.
78	Bazerbashi M, Gomez-Hassan D. Response assessment in treated brain tumors: the fundamentals. *Semin Roentgenol*. Jan 2018;53(1):37-44. doi:10.1053/j.ro.2017.11.004
79	Sanghera P, Perry J, Sahgal A, et al. Pseudoprogression following chemoradiotherapy for glioblastoma multiforme. *Can J Neurol Sci*. 2010;37(1):36-42. doi:10.1017/s0317167100009628
80	Roldan GB, Scott JN, McIntyre JB, et al. Population-based study of pseudoprogression after chemoradiotherapy in GBM. *Can J Neurol Sci*. 2009;36(5):617-22. doi:10.1017/s0317167100008131
81	Kucharczyk MJ, Parpia S, Whitton A, Greenspoon JN. Evaluation of pseudoprogression in patients with glioblastoma. *Neurooncol Pract*. Jun 2017;4(2):120-134. doi:10.1093/nop/npw021
82	Taylor C, Ekert JO, Sefcikova V, Fersht N, Samandouras G. Discriminators of pseudoprogression and true progression in high-grade gliomas: a systematic review and meta-analysis. *Sci Rep*. Aug 2 2022;12(1):13258. doi:10.1038/s41598-022-16726-x
83	Yu J, Zheng J, Xu W, et al. Accuracy of (18)F-FDOPA positron emission tomography and (18)F-FET positron emission tomography for differentiating radiation necrosis from brain tumor recurrence. *World Neurosurg*. 2018;114:e1211-e1224. doi:10.1016/j.wneu.2018.03.179
84	Kazmi F, Soon YY, Leong YH, Koh WY, Vellayappan B. Re-irradiation for recurrent glioblastoma (GBM): a systematic review and meta-analysis. *J Neurooncol*. Mar 2019;142(1):79-90. doi:10.1007/s11060-018-03064-0
85	AVASTIN (bevacizumab). Product monograph. Hoffmann-La Roche Ltd. 2018.
86	Nagpal S, Harsh G, Recht L. Bevacizumab improves quality of life in patients with recurrent glioblastoma. *Chemother Res Pract*. 2011;2011:602812. doi:10.1155/2011/602812
87	Kaka N, Hafazalla K, Samawi H, et al. Progression-free but no overall survival benefit for adult patients with bevacizumab therapy for the treatment of newly diagnosed glioblastoma: a systematic review and meta-analysis. *Cancers (Basel)*. Nov 4 2019;11(11)doi:10.3390/cancers11111723
88	Fu M, Zhou Z, Huang X, et al. Use of bevacizumab in recurrent glioblastoma: a scoping review and evidence map. *BMC Cancer*. Jun 14 2023;23(1):544. doi:10.1186/s12885-023-11043-6
89	Zhang T, Xin Q, Kang JM. Bevacizumab for recurrent glioblastoma: a systematic review and meta-analysis. *Eur Rev Med Pharmacol Sci*. Nov 2021;25(21):6480-6491. doi:10.26355/eurrev_202111_27092
90	Schritz A, Aouali N, Fischer A, et al. Systematic review and network meta-analysis of the efficacy of existing treatments for patients with recurrent glioblastoma. *Neurooncol Adv*. Jan-Dec 2021;3(1):vdab052. doi:10.1093/noajnl/vdab052
91	Ren X, Ai D, Li T, Xia L, Sun L. Effectiveness of lomustine combined with bevacizumab in glioblastoma: a meta-analysis. *Front Neurol*. 2020;11:603947. doi:10.3389/fneur.2020.603947

92	Chen Y, Guo L, Li X, Liu R, Ren C, Du S. Reduced-dose bevacizumab vs. standard-dose bevacizumab in recurrent high-grade glioma: Which one is better? A meta-analysis. *Clin Neurol Neurosurg*. Nov 2020;198:106239. doi:10.1016/j.clineuro.2020.106239
93	Azoulay M, Santos F, Shenouda G, et al. Benefit of re-operation and salvage therapies for recurrent glioblastoma multiforme: results from a single institution. *J Neurooncol*. May 2017;132(3):419-426. doi:10.1007/s11060-017-2383-2
94	Kalita O, Kazda T, Reguli S, et al. Effects of reoperation timing on survival among recurrent glioblastoma patients: a retrospective multicentric descriptive study. *Cancers (Basel)*. Apr 28 2023;15(9)doi:10.3390/cancers15092530
95	Lu VM, Jue TR, McDonald KL, Rovin RA. The survival effect of repeat surgery at glioblastoma recurrence and its trend: a systematic review and meta-analysis. *World Neurosurg*. Jul 2018;115:453-459 e3. doi:10.1016/j.wneu.2018.04.016
96	Zhao YH, Wang ZF, Pan ZY, et al. A meta-analysis of survival outcomes following reoperation in recurrent glioblastoma: time to consider the timing of reoperation. *Front Neurol*. 2019;10:286. doi:10.3389/fneur.2019.00286
97	Patel M, Au K, Easaw JC, et al. Repeat resection in recurrent glioblastoma (3rGBM) trial: a randomized care trial. *Neurochirurgie*. Apr 2022;68(3):262-266. doi:10.1016/j.neuchi.2021.09.001
98	Villani V, Prosperini L, Lecce M, et al. Recurrent glioblastoma: which treatment? A real-world study from the Neuro-oncology Unit “Regina Elena” National Cancer Institute. *Neurol Sci*. 2022;43(9):5533-5541. doi:10.1007/s10072-022-06172-y
99	McBain C, Lawrie TA, Rogozinska E, Kernohan A, Robinson T, Jefferies S. Treatment options for progression or recurrence of glioblastoma: a network meta-analysis. *Cochrane Database Syst Rev*. May 4 2021;5(1):CD013579. doi:10.1002/14651858.CD013579.pub2
100	Joseph KJ, Al-Mandhari Z, Pervez N, et al. Reirradiation after radical radiation therapy: a survey of patterns of practice among Canadian radiation oncologists. *Int J Radiat Oncol Biol Phys*. Dec 1 2008;72(5):1523-9. doi:10.1016/j.ijrobp.2008.03.048
101	Minniti G, Niyazi M, Alongi F, Navarria P, Belka C. Current status and recent advances in reirradiation of glioblastoma. *Radiat Oncol*. Feb 18 2021;16(1):36. doi:10.1186/s13014-021-01767-9
102	Vellayappan B, Kazmi F, Lim KHC, et al. Re-irradiation for recurrent glioblastoma multiforme (GBM): systematic review and meta-analysis. *International Journal of Radiation Oncology, Biology, Physics*. 2017;99(2):E114. doi:10.1016/j.ijrobp.2017.06.868
103	You WC, Lee HD, Pan HC, Chen HC. Re-irradiation combined with bevacizumab for recurrent glioblastoma beyond bevacizumab failure: survival outcomes and prognostic factors. *Sci Rep*. Jun 9 2023;13(1):9442. doi:10.1038/s41598-023-36290-2
104	Marwah R, Xing D, Squire T, Soon YY, Gan HK, Ng SP. Reirradiation versus systemic therapy versus combination therapy for recurrent high-grade glioma: a systematic review and meta-analysis of survival and toxicity. *J Neurooncol*. Sep 2023;164(3):505-524. doi:10.1007/s11060-023-04441-0
105	Stupp R, Wong ET, Kanner AA, et al. NovoTTF-100A versus physician’s choice chemotherapy in recurrent glioblastoma: a randomised phase III trial of a novel treatment modality. *Eur J Cancer*. Sep 2012;48(14):2192-202. doi:10.1016/j.ejca.2012.04.011
106	Kesari S, Ram Z, Investigators EFT. Tumor-treating fields plus chemotherapy versus chemotherapy alone for glioblastoma at first recurrence: a post hoc analysis of the EF-14 trial. *CNS Oncol*. Jul 2017;6(3):185-193. doi:10.2217/cns-2016-0049
107	Wahyuhadi J, Immadoel Haq IB, Arifianto MR, et al. Active immunotherapy for glioblastoma treatment: a systematic review and meta-analysis. *Cancer Control*. Jan-Dec 2022;29:10732748221079474. doi:10.1177/10732748221079474
108	Guo B, Zhang S, Xu L, et al. Efficacy and safety of innate and adaptive immunotherapy combined with standard of care in high-grade gliomas: a systematic review and meta-analysis. *Front Immunol*. 2023;14:966696. doi:10.3389/fimmu.2023.966696
109	Zeng YF, Wei XY, Guo QH, et al. The efficacy and safety of anti-PD-1/PD-L1 in treatment of glioma: a single-arm meta-analysis. *Front Immunol*. 2023;14:1168244. doi:10.3389/fimmu.2023.1168244
110	Reardon DA, Brandes AA, Omuro A, et al. Effect of nivolumab vs bevacizumab in patients with recurrent glioblastoma: the CheckMate 143 phase 3 randomized clinical trial. *JAMA Oncol*. Jul 1 2020;6(7):1003-1010. doi:10.1001/jamaoncol.2020.1024
111	Bagley SJ, Kothari S, Rahman R, et al. Glioblastoma clinical trials: current landscape and opportunities for improvement. *Clin Cancer Res*. 2022;28(4):594-602. doi:10.1158/1078-0432.CCR-21-2750
112	Valerius AR, Webb LM, Sener U. Novel clinical trials and approaches in the management of glioblastoma. *Curr Oncol Rep*. 2024;doi:10.1007/s11912-024-01519-4
113	Rong L, Li N, Zhang Z. Emerging therapies for glioblastoma: current state and future directions. *J Exp Clin Cancer Res*. 2022;41(1):142. doi:10.1186/s13046-022-02349-7
114	Scherm A, Ippen FM, Hau P, et al. Targeted therapies in patients with newly diagnosed glioblastoma – a systematic meta-analysis of randomized clinical trials. *Int J Cancer*. Jun 1 2023;152(11):2373-2382. doi:10.1002/ijc.34433
115	Lombardi G, De Salvo GL, Brandes AA, et al. Regorafenib compared with lomustine in patients with relapsed glioblastoma (REGOMA): a multicentre, open-label, randomised, controlled, phase 2 trial. *Lancet Oncol*. Jan 2019;20(1):110-119. doi:10.1016/S1470-2045(18)30675-2
116	STIVARGA (regorafenib). Product monograph. Bayer Inc. September 29, 2022.

117	Shi W, Roberge D, Kleinberg L, et al. Phase 3 TRIDENT study (EF-32): Tumor treating fields (TTFields; 200 kHz) concomitant with chemoradiation, and maintenance TTFields therapy/temozolomide in newly diagnosed glioblastoma. *Journal of Clinical Oncology*. 2023;41(16_suppl):TPS2083-TPS2083. doi:10.1200/JCO.2023.41.16_suppl.TPS2083
118	Tran DD, Ghiaseddin AP, Chen DD, Le SB. Final analysis of 2-THE-TOP: a phase 2 study of TTFields (Optune) plus pembrolizumab plus maintenance temozolomide (TMZ) in patients with newly diagnosed glioblastoma. *Journal of Clinical Oncology*. 2023;41(16_suppl):2024-2024. doi:10.1200/JCO.2023.41.16_suppl.2024
119	Jain KK. A critical overview of targeted therapies for glioblastoma. *Front Oncol*. 2018;8:419. doi:10.3389/fonc.2018.00419
120	Cho NS, Wong WK, Nghiemphu PL, Cloughesy TF, Ellingson BM. The future glioblastoma clinical trials landscape: early phase 0, window of opportunity, and adaptive phase I-III studies. *Curr Oncol Rep*. 2023;25(9):1047-1055. doi:10.1007/s11912-023-01433-1
121	Oppenlander ME, Wolf AB, Snyder LA, et al. An extent of resection threshold for recurrent glioblastoma and its risk for neurological morbidity. *J Neurosurg*. 2014;120(4):846-53. doi:10.3171/2013.12.JNS13184
122	Cloughesy T, Finocchiaro G, Belda-Iniesta C, et al. Randomized, double-blind, placebo-controlled, multicenter phase II study of onartuzumab plus bevacizumab versus placebo plus bevacizumab in patients with recurrent glioblastoma: efficacy, safety, and hepatocyte growth factor and O(6)-methylguanine-DNA methyltransferase biomarker analyses. *J Clin Oncol*. 2017;35(3):343-351. doi:10.1200/JCO.2015.64.7685
123	Brandes AA, Finocchiaro G, Zagonel V, et al. AVAREG: a phase II, randomized, noncomparative study of fotemustine or bevacizumab for patients with recurrent glioblastoma. *Neuro Oncol*. 2016;18(9):1304-12. doi:10.1093/neuonc/now035

Table S3. Survival outcomes with approved therapies for newly diagnosed glioblastoma [a].

Reference	Study Design	No. of Patients	Intervention	Median OS, Months (95% CI)	Median PFS, Months (95% CI)
Polonara, Aiudi et al. 2023 [1]	Retrospective	64	Tumor resection or biopsy	Supratotal resection: 24<br>GTR: 16<br>STR: 14<br>Biopsy: 10	Supratotal resection: 12.6<br>GTR: 10<br>STR: 5.9<br>Biopsy: 3.2
Zhang, Xu et al. 2020 [2]	Retrospective	26	Radiotherapy within 6 weeks after surgery vs radiotherapy ≥ 6 weeks after surgery	26.6 (18.3–34.9) vs 15.7 (9.2–22.3)	16.3 (14.7–18.0) vs 9.1 (4.7–13.4)
Stupp, Hegi et al. 2009 [3]	Randomized phase 3 trial	573	Radiotherapy with concomitant adjuvant TMZ vs radiotherapy alone	14.6 (13.2–16.8) vs 12.1 (11.2–13.0)	NA
Hegi, Diserens et al. 2005 [4]	Randomized phase 3 trial	206	Radiotherapy plus TMZ	*MGMT* promoter methylation: 21.7 (17.4–30.4)<br>Unmethylated *MGMT*: 15.3 (13.0–20.9 months)	*MGMT* promoter methylation: 10.3 (6.5–14.0)<br>Unmethylated *MGMT*: 5.3 (5.0–7.6)
Herrlinger, Tzaridis et al. 2019 [5]	Randomized, phase 3 trial	141	Lomustine plus TMZ vs TMZ alone	48.1 (32.6–NR) vs 31.4 (27.7–47.1)	16.7 (12.0–32.0) vs 16.7 (11.4–24.2)
Weller, Zeyen et al. 2022 [6]	Real-world multicentre study	321	Lomustine plus TMZ	41.0 (33.0-NR)	NA
Stupp, Taillibert et al. 2017 [7]	Randomized phase 3 study	695	TTFields plus TMZ vs TMZ alone	20.9 (19.3–22.7) vs 16.0 (14.0–18.4)	6.7 (6.1–8.1) vs 4.0 (3.8–4.4)
Ballo, Conlon et al. 2023 [8]	Meta-analysis	1,430	TTFields plus chemo vs chemo alone	22.6 (17.6–41.2) vs 17.4 (14.4–21.6)	NA

[a] This is not an exhaustive list. The most recent, largest, and high-quality evidence, wherever possible, was favored. CI, confidence interval; NA, not available; NR, not reached; OS, overall survival; PFS, progression-free survival; GTR, gross total resection; STR, subtotal resection; TMZ, temozolomide; TTFields, tumor treating fields.

Table S4. Survival outcomes with approved therapies for recurrent glioblastoma [a].

Reference	Study Design	No. of Patients	Intervention	Median OS, Months (95% CI)	Median PFS, Months (95% CI)
Azoulay, Santos et al. 2017 [9]	Retrospective	180	Repeat surgery vs no surgery	9.6 vs 5.3	NA
Kalita, Kazda et al. 2023 [10]	Retrospective	109	Repeat surgery	22.5 (19.4–29.9)	9.8 (8.5–12.5)
Oppenlander, Wolf et al. 2014 [11]; Kalita, Kazda et al. 2023 [10]	Retrospective	170	Repeat surgery	19.0	5.2
Shi, Roberge et al. 2023 [12]	Retrospective	172	Reirradiation	8.0	5.0
Villani, Prosperini et al. 2022 [13]	Real-world study	422	Chemo plus surgery, radiotherapy, or both	18 (1–152)	NA
Vellayappan, Kazmi et al. 2017 [14]; You, Lee et al. 2023 [15]	Retrospective	64	Bevacizumab and reirradiation vs supportive care	8.8 vs 3.9	NA
Nagpal, Harsh et al. 2011 [16]	Retrospective	40	Bevacizumab vs no bevacizumab	10.6 (8.9–12.3) vs 4.2 (3.0–5.3)	NA
Cloughesy, Finocchiaro et al. 2017 [17]	Randomized phase 2 trial	129	Onartuzumab plus bevacizumab vs placebo plus bevacizumab	8.8 vs 12.6	3.9 vs. 2.9
Nagpal, Harsh et al. 2011 [16]; Brandes, Finocchiaro et al. 2016 [18]	Randomized phase 2 trial	91	Bevacizumab vs fotemustine	7.3 (5.8–9.2) vs 8.7 (6.3–15.4)	NA
Stupp, Wong et al. 2012 [19]	Randomized phase 3 trial	237	TTFields vs chemo	6.6 vs 6.0	NA
Kesari, Ram et al. 2017 [20]	Post hoc study	204	TTFields plus chemo vs chemo	11.8 vs 9.2	NA

[a] This is not an exhaustive list. The most recent, largest, and high-quality evidence, wherever possible, was favored. CI, confidence interval; NA, not available; OS, overall survival; PFS, progression-free survival; TFields, tumor treating fields.

Table S5. Definitions of evidence levels [a].

Level	Definition
High-quality evidence	The participants have a lot of confidence that the available evidence supports the statement
Moderate-quality evidence	The participants believe that the available evidence probably supports the statement
Low-quality evidence	The available evidence is or might be markedly different from the effect or information reported in the statement
No evidence	There is no available evidence that supports the effect or information reported in the statement

[a] The levels were derived from the GRADE rating system. The available evidence is based on the participant's knowledge of the literature, practice experience, and other elements that can be used to infer an expert opinion on the statement.

Table S6. Ongoing [a] phase 2, 3, or 4 interventional clinical trials for glioblastoma.

NCT ID	Study Title	Status	Summary	Phase	Design
NCT05600491	A Phase III Study of Postoperative Early Temozolomide Treatment Plus Stupp Regimen for Newly	Recruiting	This study will explore the effectiveness and safety of early TMZ chemotherapy between surgery and CRT plus the standard concomitant CRT regimen.	3	Intervention model: single group Masking: none

	Diagnosed GBM Multiforme				
NCT02152982	Temozolomide With or Without Veliparib in Treating Patients With Newly Diagnosed Glioblastoma Multiforme	Active not recruiting	This randomized phase II/III trial studies how well TMZ and veliparib work compared to TMZ alone in treating patients with newly diagnosed glioblastoma multiforme. Drugs used in chemotherapy, such as TMZ, work in different ways to stop the growth of tumor cells, either by killing the cells or by stopping them from dividing or spreading. Veliparib may stop the growth of tumor cells by blocking some of the enzymes needed for cell growth. It is not yet known whether TMZ is more effective with or without veliparib in treating GBM.	2/3	Allocation: randomized Intervention model: parallel Masking: double
NCT05669820	Antisecretory Factor Glioblastoma Phase 2	Recruiting	This is a randomized, double-blind, multicentre, phase 2 study in patients with newly diagnosed glioblastoma. Participants will receive an egg powder enriched for antisecretory factors, Salovum, or a placebo egg powder daily from 2 days before concomitant radio-chemotherapy or chemotherapy until 14 days after finalization, plus during adjuvant chemotherapy. The primary aims are OS at 6 and 12 months after diagnosis.	2/3	Allocation: randomized Intervention model: parallel Masking: quadruple
NCT05095376	Testing the Addition of the Chemotherapy Drug Lomustine (Gleostine) to the Usual Treatment (Temozolomide and Radiation Therapy) for Newly Diagnosed MGMT-methylated Glioblastoma	Recruiting	This phase III trial compares the effect of adding lomustine to TMZ and radiation therapy versus TMZ and radiation therapy alone in shrinking or stabilizing newly diagnosed MGMT-methylated glioblastoma. Chemotherapy drugs, such as lomustine and TMZ, work in different ways to stop the growth of tumor cells, either by killing them, stopping them from dividing or stopping them from spreading. Radiation therapy uses high-energy photons to kill tumor cells and shrink tumors. Adding lomustine to usual treatment of TMZ and radiation therapy may help shrink and stabilize glioblastoma.	3	Allocation: randomized Intervention model: parallel Masking: none
NCT04396860	Testing the Use of the Immunotherapy Drugs Ipilimumab and Nivolumab Plus Radiation Therapy Compared to the Usual Treatment (Temozolomide and Radiation Therapy) for Newly Diagnosed MGMT Unmethylated Glioblastoma	Active not recruiting	This phase II/III trial compares the usual treatment with radiation therapy and TMZ to radiation therapy in combination with immunotherapy with ipilimumab and nivolumab in treating patients with newly diagnosed MGMT unmethylated glioblastoma. Radiation therapy uses high-energy photons to kill tumor cells and shrink tumors. Chemotherapy drugs, such as TMZ, work in different ways to stop the growth of tumor cells, either by killing the cells, preventing them from dividing or stopping them from spreading. TMZ may not work as well for the treatment of tumors that have unmethylated *MGMT*. Immunotherapy with monoclonal antibodies called immune checkpoint inhibitors, such as ipilimumab	2/3	Allocation: randomized Intervention Model: parallel Masking: none

			and nivolumab, may help the body's immune system attack the cancer, and may interfere with the ability of tumor cells to grow and spread. Immune checkpoint inhibitors may work better at the time of first diagnosis as opposed to when the tumor comes back. Giving radiation therapy with ipilimumab and nivolumab may lengthen the time without brain tumors returning or growing and may extend patients' life compared to usual treatment with radiation therapy and TMZ.		
NCT05118776	Study to Evaluate the Safety and Efficacy of ASC40 Tablets in Combination With Bevacizumab in Subjects With rGBM	Recruiting	This is a randomized, double-blind, controlled, multicentre phase III clinical trial to evaluate the safety and efficacy of ASC40 tablets combined with bevacizumab in the treatment of adult patients with recurrent glioblastoma. After standard radiotherapy and TMZ, the subject first experienced clinical recurrence or progression.	3	Allocation: randomized Intervention model: parallel Masking: quadruple
NCT03663725	Treatment Intensification With Temozolomide in Adults With a Glioblastoma	Recruiting	Due to conflicting data on the optimal moment to start TMZ chemotherapy and the impact of prolongation of the adjuvant phase with TMZ, the Association des Neuro-Oncologues d'Expression Francophone group proposes this randomized trial comparing an intensified arm (early TMZ and extended adjuvant TMZ until toxicity, progression or patient refusal) versus the classical EORTC regimen as control (RT and concomitant TMZ started 4-6 weeks after surgery followed by several adjuvant TMZ cycles strictly limited to 6) for primary GBM adult patients.	3	Allocation: randomized Intervention Model: parallel Masking: none
NCT05685004	Study of Neoantigen-specific Adoptive T Cell Therapy for Newly Diagnosed MGMT Negative Glioblastoma Multiforme (GBM)	Recruiting	This randomized study is designed to compare the combination of TVI-Brain-1 immunotherapy and standard therapy compared to standard therapy alone as a treatment for newly diagnosed MGMT unmethylated glioblastoma patients. The patients' own cancer cells collected after surgery are combined into a vaccine to produce an immune response that significantly increases the number of cancer neoantigen-specific effector T cell precursors in the patient's body. These cancer neoantigen-specific T cells are harvested from the blood, stimulated and expanded, and infused into the patient.	2/3	Allocation: randomized Intervention Model: parallel Masking: single
NCT03975829	Pediatric Long-Term Follow-up and Rollover Study	Recruiting	A rollover study to assess long-term effects in pediatric patients treated with dabrafenib and/or trametinib.	4	Intervention model: single group Masking: none
NCT00045968	Study of a Drug [DCVax®-L] to Treat Newly Diagnosed GBM Brain Cancer	Active not recruiting	The study's primary purpose is to determine the efficacy of an investigational therapy called DCVax®-L in patients with newly diagnosed GBM for whom surgery is indicated. Patients must enter screening at a	3	Allocation: randomized Intervention Model: parallel

			participating site before surgical tumor resection. Patients will receive the SoC, including radiation and Temodar therapy, and 2 out of 3 will additionally receive DCVax®-L, with the remaining one-third receiving a placebo. All patients will have the option to receive DCVax®-L in a crossover arm upon documented disease progression. (note: DCVax®-L when used for patients with brain cancer is sometimes also referred to as DCVax-Brain).		Masking: quadruple
NCT05271240	Repeated SIACI of Bevacizumab With Temozolomide and Radiation Compared to Temozolomide and Radiation Alone in Newly Diagnosed GBM	Recruiting	The investigators have completed a phase I clinical trial showing that SIACI of bevacizumab is safe up to a dose of 15mg/kg in patients with recurrent malignant glioma. Additionally, the investigators have demonstrated in a recently completed phase I/II clinical trial that SIACI bevacizumab improves the median PFS from 4-6 months to 11.5 months and OS from 12-15 months to 23 months in patients with newly diagnosed GBM. Therefore, this two-arm, randomized trial (2:1) is a follow-up study to these trials and will ask simple questions: Will this repeated SIACI treatment regimen increase PFS (primary endpoint) and OS (secondary endpoint) when compared with SoC in patients with newly diagnosed GBM? Exploratory endpoints will include adverse events, safety analysis, and QoL assessments. The investigators expect that this project will provide important information regarding the utility of repeated SIACI bevacizumab therapy for newly diagnosed GBM and may alter how these drugs are delivered to our patients.	3	Allocation: randomized Intervention model: parallel Masking: none
NCT05326464	Tofacitinib in Recurrent GBM Patients	Recruiting	This study aims to examine the effects of tofacitinib in patients with recurrent glioblastoma.	3	Intervention model: single group Masking: none
NCT05900908	Postoperative Adjuvant Therapy w/wo GammaTile + Systemic Therapy	Not yet recruiting	To compare surgical tumor removal and GammaTile therapy followed by adjuvant systemic therapy (bevacizumab or lomustine) to surgical tumor removal followed by adjuvant systemic therapy (bevacizumab or lomustine) without GammaTile therapy.	4	Allocation: randomized Intervention model: parallel Masking: single
NCT05902169	Sonocloud-9 in Association With Carboplatin Versus Standard of Care Chemotherapies (CCNU or TMZ) in Recurrent GBM	Recruiting	The brain is protected from any toxic or inflammatory molecule by the blood-brain barrier (BBB). This physical barrier is located at the level of the blood vessel walls. Because of these barrier properties, the blood vessels are also impermeable to the passage of therapeutic molecules from the blood to the brain. The development of effective treatments against glioblastoma is thus limited due to the BBB that prevents most drugs injected in the bloodstream from	3	Allocation: randomized Intervention model: parallel Masking: none

			getting into brain tissue where the tumor is seated. The SonoCloud-9 (SC9) is an investigational device using ultrasound technology and specially developed to open the BBB in the area of and surrounding the tumor. The transient opening of the BBB allows more drugs to reach the brain tumor tissue. Carboplatin is a chemotherapy that is approved to treat different cancer types alone or in combination with other drugs and has been used in the treatment of glioblastoma. Despite its proven efficacy in the laboratory on glioblastoma cells, carboplatin does not readily cross the BBB in humans. A clinical trial has shown that in combination with the SonoCloud-9, more carboplatin can reach the brain tumor tissue. The objective of the proposed trial is to show that the association - carboplatin with the SonoCloud-9 - will increase the drug's efficacy in patients with recurrent glioblastoma.		
NCT05318612	Effectiveness of MR-guided LITT Therapy in Irresectable Glioblastoma (EMITT)	Active not recruiting	This study aims to investigate the cost-effectiveness of Laser Interstitial Thermal Therapy in primary irresectable glioblastoma. The primary aim of this project is to examine whether laser therapy combined with CRT improves OS without compromising QoL in comparison with CRT alone in patients with primary irresectable glioblastoma.	3	Allocation: randomized Intervention model: parallel Masking: none
NCT05100641	AV-GBM-1 vs Control as Adjunctive Therapy Following Surgery and RT/TMZ in Newly Diagnosed GBM	Not yet recruiting	This is a multicentre, double-blind, 2:1 randomized phase III trial to determine whether the addition of AV-GBM-1, a therapeutic, patient-specific dendritic cell vaccine, to standard therapy increases OS of patients with a recent diagnosis of primary GBM.<br><br>The intent is to enroll approximately 726 patients for tumor collection to enroll 690 who are eligible for treatment at the time of randomization and who have granted consent for participation. Because of the lack of toxicity, there are no restrictions related to performance status or blood tests at the time of treatment. The key endpoint is OS from date of first injection after RT/TMZ; secondary endpoints are PFS from date of first injection and OS and PFS from date of randomization before RT/TMZ. The date of PFS will be determined by the principal investigator at each site.	3	Allocation: randomized Intervention model: parallel Masking: quadruple
NCT05904119	Lomustine With and Without Reirradiation for First Progression of Glioblastoma: A	Recruiting	Despite comprehensive multimodal treatment of newly diagnosed glioblastoma, almost all patients suffer from tumor relapse. Currently, no SoC exists to treat these tumor relapses. Treatment options include repeated	3	Allocation: randomized Intervention model: parallel Masking: none

	Randomized Phase III Study		surgery (if feasible), systemic therapy (bevacizumab, lomustine, TMZ rechallenge), reirradiation and best supportive care. Currently, the superiority of combined chemoradiation versus chemotherapy alone remains unproven. Given that lomustine is the standard chemotherapeutic agent for the treatment of recurrent glioblastoma in Europe and the unclear efficacy of reirradiation, we want to explore whether combining lomustine and reirradiation may be a better treatment than lomustine alone. The results of the prospective randomized trial proposed here should demonstrate a significant improvement in OS when lomustine is combined with reirradiation in patients with recurrent glioblastoma compared to lomustine alone without adversely affecting quality of survival. The trial will be stopped based on OS in a preplanned futility and efficacy interim analysis.		
NCT02017717	A Study of the Effectiveness and Safety of Nivolumab Compared to Bevacizumab and of Nivolumab With or Without Ipilimumab in Glioblastoma Patients	Active not recruiting	The purpose of the study is to compare the efficacy and safety of nivolumab administered alone versus bevacizumab in patients diagnosed with recurrent glioblastoma and to evaluate the safety and tolerability of nivolumab administered alone or in combination with ipilimumab in patients with different lines of GBM therapy.	3	Allocation: randomized Intervention model: parallel Masking: none
NCT03008148	Phase II/III Trial of CCRT With or Without JP001 for Newly Diagnosed GBM	Recruiting	This is a multicentre, phase II/III, open-label, randomized, parallel and standard chemoradiation-controlled study where eligible subjects will be randomized at 1:1 ratio to receive control treatment or study treatment. The primary objective of this trial is to evaluate the effect of add-on JP001 to standard chemoradiation in increasing OS on newly diagnosed GBM patients.	2/3	Allocation: randomized Intervention model: parallel Masking: none
NCT06105619	A Study of PLB1001 Enteric Capsules in the Treatment of sGBM/IDH-Mutant Glioblastoma Patients With the ZM Fusion Gene (FUGEN)	Active not recruiting	The goal of this clinical trial is to evaluate the safety and efficacy of PLB1001 Enteric Capsules in the treatment of PTPRZ1-MET fusion gene-positive recurrent secondary glioblastoma. The main questions it aims to answer are:<br><br>1. To evaluate OS in the treatment of secondary glioblasts with positive recurrence of PTPRZ1-MET fusion gene by PLB1001 Enteric Capsules.<br>2. To evaluate if it is safe and tolerant in the treatment of secondary glioblasts with positive recurrence of PTPRZ1-MET fusion gene by PLB1001 Enteric Capsules.	2/3	Allocation: randomized Intervention model: parallel Masking: none
NCT04250922	LAM561 With RT and TMZ for Adults With Glioblastoma	Recruiting	The proposed phase IIB/III randomized, double-blind, placebo-controlled trial in subjects with newly diagnosed primary glioblastoma multiforme aims to compare	2/3	Allocation: randomized Intervention model: parallel

			the efficacy and safety of LAM561 versus placebo, given with SoC therapy of radiation therapy plus TMZ, followed by an adjuvant treatment of 6-month period of TMZ and then LAM561 or placebo in monotherapy.		Masking: triple
NCT05342883	GammaTile and Stupp in Newly Diagnosed GBM	Recruiting	SoC postoperative chemoradiation for patients with newly diagnosed GBM does not routinely provide durable local control or prolonged OS. As discussed above, it seems unlikely that patient outcomes will be significantly improved with radiation dose escalation given at the time of the EBRT boost. However, as most failures are local, improving LC could potentially improve the OS of patients. To do this, we propose a shift in the traditional radiation paradigm. This study will assess the feasibility and tolerability of adding GT radiation therapy as an upfront boost at the time of maximum safe resection, along with the backbone of the current SoC approach, concomitant and adjuvant TMZ +/- TTF, for patients with newly diagnosed GBM. GT, a novel brain brachytherapy device utilizing Cs-131 embedded in bioresorbable collagen tiles, offers a more sophisticated carrier and a shorter half-life radioisotope, Cs-131. This device allows for radiation initiation at an earlier time, more rapid dose delivery, and possibly more effective tumor control, particularly for rapidly proliferating tumors such as GBM. Two prospective studies have demonstrated the safety and efficacy of reirradiation with GT in patients with recurrent GBM. This single-arm, open-label phase 4 study aims to determine the feasibility and tolerability of treating patients with GammaTile in combination with the Stupp protocol and how to proceed with testing this treatment in a future, larger, randomized clinical study. The study aims to demonstrate that the use of GammaTile at the time of surgery is well tolerated and does not delay the start of the Stupp protocol. Efficacy outcomes (e.g., LC, OS, PFS) will also be described.	4	Intervention model: single group Masking: none
NCT02685605	Intraoperative Radiotherapy in Newly Diagnosed Glioblastoma Multiforme	Recruiting	INTRAGO II resembles a multicentric, prospective, randomized, 2-arm, open-label clinical phase III trial, which tests if the median PFS of patients with newly diagnosed GBM can be improved by the addition of intraoperative radiotherapy to standard radiochemotherapy.	3	Allocation: randomized Intervention model: parallel Masking: none
NCT05439278	Conventional Versus Hypofractionated Radiotherapy With Temozolomide in Elderly Glioblastoma	Not yet recruiting	In newly diagnosed glioblastoma patients aged 70 years or older who are suitable for concurrent TMZ, the optimal dose of radiation therapy is controversial. The purpose of this study is to compare	3	Allocation: randomized Intervention model: parallel Masking: none

			conventional radiotherapy of 60 Gy (6 weeks) versus hypofractionated radiotherapy of 40 Gy (3 weeks) in terms of OS as the primary endpoint along with PFS, toxicity, QoL, and prognostic biomarkers.		
NCT03548571	Dendritic Cell Immunotherapy Against Cancer Stem Cells in Glioblastoma Patients Receiving Standard Therapy	Recruiting	Open, randomized study of a trivalent dendritic cell therapy compared to standard therapy in primary treated patients with IDH-wildtype, MGMT-promotor methylated glioblastoma. The IMP is dendritic cells transfected with mRNA of survivin, hTERT of autologous tumor stem cells derived from tumorspheres.	2/3	Allocation: randomized Intervention model: parallel Masking: none
NCT03776071	A Trial of Enzastaurin Plus Temozolomide During and Following Radiation Therapy in Patients With Newly Diagnosed Glioblastoma With or Without the Novel Genomic Biomarker, DGM1	Active not recruiting	This will be a randomized, double-blind, placebo-controlled, multicentre phase 3 study. Approximately 300 subjects with newly diagnosed glioblastoma who meet all eligibility criteria will be enrolled.	3	Allocation: randomized Intervention model: parallel Masking: triple
NCT02761070	Bevacizumab Alone Versus Dose-dense Temozolomide Followed by Bevacizumab for Recurrent Glioblastoma, Phase III	Active not recruiting	This phase III study aims to evaluate the superiority of ddTMZ followed by bevacizumab at ddTMZ failure for glioblastoma at first recurrence or progression, compared to bevacizumab alone.	3	Allocation: randomized Intervention model: parallel Masking: none
NCT04536649	Proton and Heavy Ion Beam Radiation vs. Photon Beam Radiation for Newly Diagnosed Glioblastoma	Not yet recruiting	This multicentre prospective phase 3 clinical trial explores the efficacy and side effects of standard-dose photon radiation versus standard-dose proton radiation versus carbon-ion boost plus standard proton radiation for newly diagnosed glioblastoma. The patients enrolled will be randomly allocated 1:1:1 to three groups: Control Group, standard-dose photon radiotherapy; Study Group A, standard-dose proton radiotherapy; Study Group B, standard-dose proton radiotherapy plus induction carbon-ion radiotherapy boost. The primary endpoint is OS.	3	Allocation: randomized Intervention model: parallel Masking: none
NCT03243461	International Cooperative Phase III Trial of the HIT-HGG Study Group (HIT-HGG-2013)	Recruiting	The HIT-HGG-2013 trial offers an innovative, high-quality diagnostics and science program for children and adolescents >3 years suffering from one of the following types of high-grade gliomas: glioblastoma WHO grade IV, diffuse midline glioma histone 3 K27M mutated WHO grade IV, anaplastic astrocytoma WHO grade III, diffuse intrinsic pontine glioma, and gliomatosis cerebri.<br><br>In addition to standard treatment (radiotherapy and TMZ), the effect of valproic acid, which is traditionally used for the treatment of seizure disorder, will be investigated. The aim of the trial will be to	3	Intervention model: single group Masking: none

			investigate whether this drug may increase the effects of radio- and chemotherapy, resulting in a better survival of the treated patients. Scientific studies provided evidence for the antitumoral effects of valproic acid: the drug seems to be a so-called histone deacetylase inhibitor (HDAC inhibitor), controlling important genetic processes of tumor growth.<br><br>The aim of the HIT-HGG-2013 trial will be to compare the effects of Valproic acid with data from the HIT-HGG-2007 trial (children and adolescents with the same diseases, only treated with simultaneous TMZ radiochemotherapy).		
NCT03970447	A Trial to Evaluate Multiple Regimens in Newly Diagnosed and Recurrent Glioblastoma	Recruiting	GBM adaptive, global, innovative learning environment (GBM AGILE) is an international, seamless phase II/III response adaptive randomization platform trial designed to evaluate multiple therapies in newly diagnosed and recurrent GBM.	2/3	Allocation: randomized Intervention model: sequential Masking: none
NCT02667587	An Investigational Immuno-therapy Study of Temozolomide Plus Radiation Therapy With Nivolumab or Placebo for Newly Diagnosed Patients With Glioblastoma (GBM, a Malignant Brain Cancer)	Active not recruiting	The purpose of this study is to evaluate patients with glioblastoma that is *MGMT*-methylated (the *MGMT* gene is altered by a chemical change). Patients will receive TMZ plus radiation therapy. They will be compared to patients receiving nivolumab in addition to TMZ plus radiation therapy.	3	Allocation: randomized Intervention model: parallel Masking: triple
NCT05235737	The Assessment of Immune Response in Newly Diagnosed Glioblastoma Patients Treated With Pembrolizumab	Recruiting	To evaluate the short-term and longer-term safety, tolerability, and effectiveness of neoadjuvant and adjuvant pembrolizumab on top of standard therapy (Stupp protocol) in patients with GBM. Randomized comparison of safety, tolerability, and clinical efficacy of (1) neoadjuvant and adjuvant pembrolizumab (on top of Stupp protocol, n=12 patients), (2) neoadjuvant pembrolizumab (on top of Stupp protocol, n=12 patients), and (3) SoC (Stupp protocol only, n=12 patients). Immuno-PET examination will be performed before and after surgery in all patients.	4	Allocation: randomized Intervention model: parallel Masking: none

[a] Studies that are not yet recruiting, recruiting, or active not recruiting are listed. CRT, chemoradiotherapy; ddTMZ, dose-dense temozolomide; GBM, glioblastoma multiforme; OS, overall survival; PFS, progression-free survival; QoL, quality of life; SIACI, superselective intra-arterial cerebral infusion; SoC, standard of care; TMZ, temozolomide.

References

1. Polonara, G.; Aiudi, D.; Iacoangeli, A.; Raggi, A.; Ottaviani, M.M.; Antonini, R.; Iacoangeli, M.; Dobran, M. Glioblastoma: A retrospective analysis of the role of the maximal surgical resection on overall survival and progression-free survival. *Biomedicines* **2023**, *11*, 739. https://doi.org/10.3390/biomedicines11030739.
2. Zhang, M.; Xu, F.; Ni, W.; Qi, W.; Cao, W.; Xu, C.; Chen, J.; Gao, Y. Survival impact of delaying postoperative chemoradiotherapy in newly-diagnosed glioblastoma patients. *Transl. Cancer Res.* **2020**, *9*, 5450–5458. https://doi.org/10.21037/tcr-20-1718.
3. Stupp, R.; Hegi, M.E.; Mason, W.P.; van den Bent, M.J.; Taphoorn, M.J.; Janzer, R.C.; Ludwin, S.K.; Allgeier, A.; Fisher, B.; Belanger, K.; et al. Effects of radiotherapy with concomitant and adjuvant temozolomide versus radiotherapy alone on survival in glioblastoma in a randomised phase III study: 5-year analysis of the EORTC-NCIC trial. *Lancet Oncol.* **2009**, *10*, 459–466. https://doi.org/10.1016/S1470-2045(09)70025-7.
4. Hegi, M.E.; Diserens, A.C.; Gorlia, T.; Hamou, M.F.; de Tribolet, N.; Weller, M.; Kros, J.M.; Hainfellner, J.A.; Mason, W.; Mariani, L.; et al. MGMT gene silencing and benefit from temozolomide in glioblastoma. *N. Engl. J. Med.* **2005**, *352*, 997–1003. https://doi.org/10.1056/NEJMoa043331.
5. Herrlinger, U.; Tzaridis, T.; Mack, F.; Steinbach, J.P.; Schlegel, U.; Sabel, M.; Hau, P.; Kortmann, R.D.; Krex, D.; Grauer, O.; et al. Lomustine-temozolomide combination therapy versus standard temozolomide therapy in patients with newly diagnosed glioblastoma with methylated MGMT promoter (CeTeG/NOA-09): A randomised, open-label, phase 3 trial. *Lancet* **2019**, *393*, 678–688. https://doi.org/10.1016/S0140-6736(18)31791-4.
6. Weller, J.; Zeyen, T.; Schlegel, U.; Lazaridis, L.; Werner, J.-M.; Onken, J.; Zeiner, P.; Drexler, R.; Hau, P.; Seidel, C.; et al. Lomustine/temozolomide chemotherapy for newly diagnosed MGMT-methylated IDHwt glioblastoma according to CeTeG/NOA–09: Real-world experience in a multicenter cohort. *Neuro-Oncology* **2022**, *24*, vii71. https://doi.org/10.1093/neuonc/noac209.274.
7. Stupp, R.; Taillibert, S.; Kanner, A.; Read, W.; Steinberg, D.; Lhermitte, B.; Toms, S.; Idbaih, A.; Ahluwalia, M.S.; Fink, K.; et al. Effect of Tumor-Treating Fields plus maintenance temozolomide vs maintenance temozolomide alone on survival in patients with glioblastoma: A randomized clinical trial. *JAMA* **2017**, *318*, 2306–2316. https://doi.org/10.1001/jama.2017.18718.
8. Ballo, M.T.; Conlon, P.; Lavy-Shahaf, G.; Kinzel, A.; Vymazal, J.; Rulseh, A.M. Association of tumor treating fields (TTFields) therapy with survival in newly diagnosed glioblastoma: A systematic review and meta-analysis. *J. Neurooncol.* **2023**, *164*, 1–9. https://doi.org/10.1007/s11060-023-04348-w.
9. Azoulay, M.; Santos, F.; Shenouda, G.; Petrecca, K.; Oweida, A.; Guiot, M.C.; Owen, S.; Panet-Raymond, V.; Souhami, L.; Abdulkarim, B.S. Benefit of re-operation and salvage therapies for recurrent glioblastoma multiforme: Results from a single institution. *J. Neurooncol.* **2017**, *132*, 419–426. https://doi.org/10.1007/s11060-017-2383-2.
10. Kalita, O.; Kazda, T.; Reguli, S.; Jancalek, R.; Fadrus, P.; Slachta, M.; Pospisil, P.; Krska, L.; Vrbkova, J.; Hrabalek, L.; et al. Effects of reoperation timing on survival among recurrent glioblastoma patients: A retrospective multicentric descriptive study. *Cancers (Basel)* **2023**, *15*, 2530. https://doi.org/10.3390/cancers15092530.
11. Oppenlander, M.E.; Wolf, A.B.; Snyder, L.A.; Bina, R.; Wilson, J.R.; Coons, S.W.; Ashby, L.S.; Brachman, D.; Nakaji, P.; Porter, R.W.; et al. An extent of resection threshold for recurrent glioblastoma and its risk for neurological morbidity. *J. Neurosurg.* **2014**, *120*, 846–853. https://doi.org/10.3171/2013.12.JNS13184.
12. Shi, W.; Roberge, D.; Kleinberg, L.; Jeyapalan, S.A.; Goldlust, S.A.; Nagpal, S.; Lustgarten, L.; Combs, S.E.; Nishikawa, R.; Reardon, D.A.; et al. Phase 3 TRIDENT study (EF-32): Tumor treating fields (TTFields; 200 kHz) concomitant with chemoradiation, and maintenance TTFields therapy/temozolomide in newly diagnosed glioblastoma. *J. Clin. Oncol.* **2023**, *41*, TPS2083–TPS2083. https://doi.org/10.1200/JCO.2023.41.16_suppl.TPS2083.
13. Villani, V.; Prosperini, L.; Lecce, M.; Tanzilli, A.; Farneti, A.; Benincasa, D.; Telera, S.; Marucci, L.; Piludu, F.; Pace, A. Recurrent glioblastoma: Which treatment? A real-world study from the Neuro-oncology Unit "Regina Elena" National Cancer Institute. *Neurol. Sci.* **2022**, *43*, 5533–5541. https://doi.org/10.1007/s10072-022-06172-y.
14. Vellayappan, B.; Kazmi, F.; Lim, K.H.C.; Yeo, T.T.; Wong, A.; Soon, Y.Y.; Koh, W.Y. Re-irradiation for recurrent glioblastoma multiforme (GBM): Systematic review and meta-analysis. *Int. J. Radiat. Oncol. Biol. Phys.* **2017**, *99*, E114. https://doi.org/10.1016/j.ijrobp.2017.06.868.
15. You, W.C.; Lee, H.D.; Pan, H.C.; Chen, H.C. Re-irradiation combined with bevacizumab for recurrent glioblastoma beyond bevacizumab failure: Survival outcomes and prognostic factors. *Sci. Rep.* **2023**, *13*, 9442. https://doi.org/10.1038/s41598-023-36290-2.
16. Nagpal, S.; Harsh, G.; Recht, L. Bevacizumab improves quality of life in patients with recurrent glioblastoma. *Chemother. Res. Pract.* **2011**, *2011*, 602812. https://doi.org/10.1155/2011/602812.

17. Cloughesy, T.; Finocchiaro, G.; Belda-Iniesta, C.; Recht, L.; Brandes, A.A.; Pineda, E.; Mikkelsen, T.; Chinot, O.L.; Balana, C.; Macdonald, D.R.; et al. Randomized, double-blind, placebo-controlled, multicenter phase II study of onartuzumab plus bevacizumab versus placebo Plus bevacizumab in patients with recurrent glioblastoma: Efficacy, safety, and hepatocyte growth factor and O(6)-methylguanine-DNA methyltransferase biomarker analyses. *J. Clin. Oncol.* **2017**, *35*, 343–351. https://doi.org/10.1200/JCO.2015.64.7685.
18. Brandes, A.A.; Finocchiaro, G.; Zagonel, V.; Reni, M.; Caserta, C.; Fabi, A.; Clavarezza, M.; Maiello, E.; Eoli, M.; Lombardi, G.; et al. AVAREG: A phase II, randomized, noncomparative study of fotemustine or bevacizumab for patients with recurrent glioblastoma. *Neuro Oncol.* **2016**, *18*, 1304–1312. https://doi.org/10.1093/neuonc/now035.
19. Stupp, R.; Wong, E.T.; Kanner, A.A.; Steinberg, D.; Engelhard, H.; Heidecke, V.; Kirson, E.D.; Taillibert, S.; Liebermann, F.; Dbaly, V.; et al. NovoTTF-100A versus physician's choice chemotherapy in recurrent glioblastoma: A randomised phase III trial of a novel treatment modality. *Eur. J. Cancer* **2012**, *48*, 2192–2202. https://doi.org/10.1016/j.ejca.2012.04.011.
20. Kesari, S.; Ram, Z.; Investigators, E.F.T. Tumor-treating fields plus chemotherapy versus chemotherapy alone for glioblastoma at first recurrence: A post hoc analysis of the EF-14 trial. *CNS Oncol.* **2017**, *6*, 185–193. https://doi.org/10.2217/cns-2016-0049.
